# Germline Pathogenic Variants Identified by Targeted Next-Generation Sequencing of Susceptibility Genes in Pheochromocytoma and Paraganglioma

**DOI:** 10.15586/jkcvhl.v8i1.171

**Published:** 2021-03-13

**Authors:** Sinem Yalcintepe, Hakan Gurkan, Fatma Nur Korkmaz, Selma Demir, Engin Atli, Damla Eker, Hazal Sezginer Guler, Drenusha Zhuri, Emine Ikbal Atli, Semra Ayturk Salt, Mustafa Sahin, Sibel Guldiken

**Affiliations:** 1 Department of Medical Genetics, Faculty of Medicine, Trakya University, Edirne, Turkey;; 2 Department of Endocrinology and Metabolism, Faculty of Medicine, Ankara University, Ankara, Turkey;; 3 Division of Endocrinology, Department of Internal Medicine, Faculty of Medicine, Trakya University, Edirne, Turkey

**Keywords:** paraganglioma, pheochromocytoma, targeted sequencing, susceptibility genes

## Abstract

The aim of this study was to evaluate germline variant frequencies of pheochromocytoma and paraganglioma targeted susceptibility genes with next-generation sequencing method. Germline DNA from 75 cases were evaluated with targeted next-generation sequencing on an Illumina NextSeq550 instrument. *KIF1B, RET, SDHB, SDHD, TMEM127*, and *VHL* genes were included in the study, and Sanger sequencing was used for verifying the variants. The pathogenic/likely pathogenic variants were in the *VHL, RET, SDHB*, and *SDHD* genes, and the diagnosis rate was 24% in this study. Three different novel pathogenic variants were determined in five cases. This is the first study from Turkey, evaluating germline susceptibility genes of pheochromocytoma and paraganglioma with a detection rate of 24% and three novel variants. All patients with pheochromocytoma and paraganglioma need clinical genetic testing with expanded targeted gene panels for higher diagnosis rates.

## Introduction

Paraganglioma and pheochromocytoma are defined as neurogenic tumors arising from paraganglial cells found in neuroendocrine tissues (1). Paraganglionic tissues, on the other hand, are specialized cell groups and are generally found scattered throughout the body. These cells are mostly located in the adrenal medulla, aorta, vascular wall, heart, prostate, and ovaries (2). They are groups of cells in close proximity to the sympathetic and parasympathetic nervous system. Tumors originating from chromaffin cell groups are called pheochromocytomas. (3). Paragangliomas do not generally contain epinephrine and are not stained with chromium salts. Therefore, these tumors are considered chromaffin negative. These paraganglial cell groups are found in the carotid, jugular glomus, aorticopulmonary glomus, vagus, and ciliary glomus where chemoreceptors are found in the body (1).

Von Hippel–Lindau (VHL) Syndrome is a hereditary cancer syndrome involving more than one system, characterized by benign or malignant tumors and cystic lesions (4). Hemangioblastomas, renal cysts and kidney cancer, pheochromocytoma, pancreatic cysts and neuroendocrine tumors, endolymphatic sac tumors, and epididymal and ligamentum latum cysts may be seen in the brain, spinal cord, and retina (5). Retinal hemangioblastomas are usually the first sign of the disease and cause vision loss. Up to 70% of the affected individuals can be diagnosed with retinal hemangioblastomas detected around the age of 25 (6, 7).

Pheochromocytoma associated with a pathogenic variant occurs at a younger age than pheochromocytoma cases without any mutation (8). Although there is no clear age limit, having been diagnosed younger than 30–45 may indicate the presence of a mutation. *SDHA, SDHAF2, SDHB, SDHC, SDHD, MAX*, and *TMEM127* genes are offered to be analyzed for the diagnosis of paraganglioma/pheochromocytoma (PGL/PCC) syndromes (9). For differential diagnosis, *NF1, VHL, RET*, and *EPAS1* genes should be analyzed (10). In this study, a custom panel of

*KIF1B, RET, SDHB, SDHD, TMEM127*, and *VHL* genes were analyzed with a targeted gene panel using next-generation sequencing method for rapid and cost-effective diagnosis and differential diagnosis in patients with PGL/PCC. Only these six genes could be analyzed, as this study was planned retrospectively and this custom panel was used between the years 2013 and 2020 in our department; this custom panel was designed firstly in 2013 with only six genes. To our knowledge, this is the first study presenting germline pathogenic/likely pathogenic variants of paraganglioma and pheochromocytoma patients from Turkey.

## Material and Methods

### Patients

Seventy-five cases with a pre-diagnosis of PGL/PCC were examined in the Endocrinology Clinic and referred to the Medical Genetics clinic for molecular testing from August 2013 through November 2020. Cases were included for having high levels of blood or urinary catecholamines, metanephrines, methoxytyramine, or chromogranin A, and/or a suspicion of PGL/PCC on imaging tests, and/or personal or family history of PGL/PCC in our study. Pediatric cases and/or adult cases who have not had any imaging tests due to pregnancy, breastfeeding, claustrophobia, medical conditions, serious illness, and acute or chronic renal insufficiency were excluded from this study.

The cases had no relation except cases 4 and 5 who are brothers (Table 1).

**Table 1:** Demographic features, phenotypes, and genotypes of the cases with pathogenic/likely pathogenic variants detected in our study.

Case	Age/Gender	Phenotype	Affected family member	Gene	Nucleotide change	Protein	dbSNP or HGMD ID
1 FA	40/F	Pheochromocytoma	Unknown	*VHL*	NM_000551.4:c.583C>T	p.(Gln195Ter)	rs5030825
2 MA	35/M	Pheochromocytoma (Bilateral)	Father, sister	*VHL*	NM_000551.4:c.481C>T	p.(Arg161Ter)	rs5030818
3 MAU	27/M	Pheochromocytoma (Bilateral surrenal—metastatic lymph nodes)	Unknown	*VHL*	NM_000551.4:c.277G>A	p.(Gly93Ser)	rs5030808
4 AGY	29/M	Pheochromocytoma (Bilateral renal and eyes, pancreatic cysts))	Mother, brother (Case 5)	*VHL*	NM_000551.4:c.202T>C	p.(Ser68Pro)	CM073416
5 AY	32/M	Pheochromocytoma (Right kidney-clear cell carcinoma)	Mother, brother (Case 4)	*VHL*	NM_000551.4:c.202T>C	p.(Ser68Pro)	CM073416
6 MD	33/M	Paraganglioma, liver cysts	Cousin	*VHL*	NM_000551.4:c.202T>C	p.(Ser68Pro)	CM073416
7 MG	42/M	MEN2A (Pheochromocytoma, medullary thyroid cancer, hyperparathyroid)	No history	*RET*	NM_020975.6:c.1832G>A	p.(Cys611Tyr)	rs377767397
8 AB	62/M	Medullary thyroid cancer	No history	*RET*	NM_020975.6:c.1946C>T	p.(Ser649Leu)	rs148935214
9 UB	47/F	Right thyroid lobe nodule, high serum calcitonin level	No history	*RET*	NM_020975.6:c.2370G>T	p.(Leu790Phe)	rs75030001
10 FB	28/F	Medullary thyroid cancer	Unknown	*RET*	NM_020975.6:c.1901G>A	p.(Cys634Tyr)	rs75996173
11 HG	59/F	Pheochromocytoma	Unknown	*RET*	NM_020975.6:c.1900T>C	p.(Cys634Arg)	rs75076352
12 MK	22/M	Paraganglioma	Unknown	*SDHB*	NM_003000.3:c.508dupT	p.(Tyr170LeufsTer9)	novel
13 BB	15/M	Paraganglioma	No history	*SDHB*	NM_003000.3:c.298T>C	p.(Ser100Pro)	CM056397
14 HT	24/M	Tumor located posterior to the liver segment	No history	*SDHB*	NM_003000.3:c.262A>C	p.(Thr88Pro)	novel
15 AC	18/M	Prevertebral paraganglioma, hypertrophic cardiomyopathy	Unknown	*SDHB*	NM_003000.3:c.262A>C	p.(Thr88Pro)	novel
16 YB	30/M	Pheochromocytoma (Right surrenal)	No history	*SDHD*	NM_003002.4:c.147dupA	p.(His50ThrfsTer19)	rs876659130
17 BS	82/F	Paraganglioma, medullary thyroid cancer	No history	*SDHD*	NM_003002.4:c.326A>G	p.(Gln109Arg)	novel
18 MY	24/F	Pheochromocytoma (Right surrenal)	Grandmother-Hypertension	*SDHD*	NM_003002.4:c.326A>G	p.(Gln109Arg)	novel

dbSNP: database of single nucleotide polymorphisms, HGMD: The Human Gene Mutation Database.

The written informed consent forms were obtained from the patients or from their legal guardians. This study is approved by the Ethical Committee of our university with the number 2021/46 and performed in consonance with the principles of the Declaration of Helsinki.

### Molecular analysis

Genomic DNA was isolated from peripheral blood samples with ethylenediaminetetraacetic acid tetrasodium salt dihydrate (EDTA) of the patients by using EZ1 DNA Investigator Kit (Qiagen, Hilden, Germany). Primary quality control of the isolated DNA samples was performed using NanoDrop (Thermo Fisher Scientific, Waltham, MA), and samples having A260/280 values between 1.8 and 2.0 were included in the study.

QIAseq Targeted DNA Panel (Qiagen, Hilden, Germany) was performed to analyze six genes (*KIF1B, RET, SDHB, SDHD, TMEM127*, and *VHL*). Libraries were prepared according to the manufacturer’s instructions. Quality control of the prepared libraries was applied with Qubit dsDNA BR Assay system (Invitrogen, Carlsbad, CA). Fastq files were performed on Illumina NextSeq550 (Illumina Inc., San Diego, CA, ABD). Libraries covering the target genes were prepared according to the QIAseq Targeted DNA Panel protocol (Qiagen, Hilden, Germany). Following the target enrichment process, libraries were sequenced on the Illumina NextSeq550 (Illumina Inc., San Diego, CA, ABD). QCI analysis (Qiagen, Hilden, Germany) was used for Quality control and ordering Variant Call Format file. Variant analysis was performed with Ingenuity software (Qiagen, Hilden, Germany).

For verifying the variants and segregation analysis, primers were designed for all needed regions, and Sanger sequencing was performed using an ABI 3130 (Applied Biosystems, USA) capillary electrophoresis system.

### Variant classification

ACMG-2015 (American College of Medical Genetics) (11) guidelines were followed for the classification of all the variants, and recommendations of the Human Genome Variation Society (12) were followed to describe the novel variants. ClinVar (13), HGMD-Professionel 2020 database, and literature information were considered for collecting the information about known variants.

## Results

Six different germline genes (*KIF1B, RET, SDHB, SDHD, TMEM127*, and *VHL*) were analyzed in 75 cases with a pre-diagnosis of PGL/PCC. These genes were covered by 241 amplicons with Custom QIAseq Paraganglioma Panel (CDHS-17364Z-241). Two hundred and thirty eight of 241 amplicons (99%) yielded sequence reads, with a mean depth of 950 × per amplicon and sample.

The cases had a mean age of 40.4 (38 female cases with a mean age of 46.5; 37 male cases with a mean age of 34.1). Five of the patients (27.7%) had an affected family member.

Eighteen cases (24%) had pathogenic/likely pathogenic variants in our study. Six cases had pathogenic/likely pathogenic variants in *VHL* gene-four different variants. Five cases had five different pathogenic/likely pathogenic variants in *RET* gene. Four cases had three different pathogenic/likely pathogenic variants (two novel variants) in *SDHB* gene. Three cases had two different pathogenic/likely pathogenic variants and one novel variant in *SDHD* gene. We detected three different novel pathogenic/likely pathogenic variants in this study.

## Discussion

In the current study, we designed a targeted NGS assay for susceptibility genes to analyze the pheochromocytoma and/or paraganglioma. *SDHB, SDHD, RET*, and *VHL* genes showed pathogenic variants in our study. Five of 18 cases (27.7%) were diagnosed with a novel pathogenic/likely pathogenic variant. Prevalence of germline mutations is an important consideration for offering molecular diagnostics.

In this study, six cases was diagnosed with *VHL* pathogenic variants and the younger patient with *VHL* pathogenic/likely pathogenic variant was 27 years old (Table 1, Case 3). He was diagnosed with metastatic bilateral surrenal adenomas. A study analyzing 86 unselected PGL/PCC tumor samples reported that two pathogenic variants were detected in *VHL* gene (14). *VHL* gene was also reported in 19.1% of the cases as the reason for bilateral pheochromocytoma (15). Fifty percent of our *VHL* cases had bilateral pheochromocytoma, one case had also eye involvement and pancreatic cysts, and one case had liver cysts (Table 1).

In the current study, five cases had different pathogenic *RET* variants with different clinical presentations as pheochromocytoma, medullary thyroid cancer, and thyroid nodules. The ages of these cases were between 28 and 62 years. Medullary thyroid cancer is a rare tumor originating from the parafollicular C cells of the thyroid, and it is characterized by *RET* proto-oncogene mutation in almost all hereditary cases and more than 40% of sporadic cases (16). In addition to clinical findings in medullary thyroid cancer evaluation, *RET* proto-oncogene mutation screening is highly recommended to determine genotype-phenotype risks and to distinguish between sporadic and familial cases in terms of maintenance of the disease. *RET* polymorphic alleles were reported as an additive effect on the estimated risk of age-related pheochromocytoma penetrance in MEN2 patients (17). Pheochromocytoma in MEN2 patients is usually reported as bilateral and rarely to be metastatic (18). It was reported that analysis of patients with *RET* 634 mutations with and without pheochromocytoma showed that pheochromocytoma was not associated with a more advanced stage of medullary thyroid cancer at diagnosis or a shorter survival (18). We detected codon 634 mutations in two cases (40%) of five *RET*-mutated cases—C634Y-mutated case had medullary thyroid cancer, and C634R-mutated case had pheochromocytoma. Similarly with the literature, our cases had mostly codon 634 mutations. As a limitation of this single-center study, the current cohort was limited. Codon 634 mutations were reported as the most frequent *RET* mutations in Brazil (19). C634Y (63.4%), C634R (28.2%), C634W (3.8%), C634G (3.1%), C634S (1.1%), and C634F (0.4%) mutations were reported in the same study (19). Codon 634 mutations were considered to be associated with an aggressive clinical course (20).

A 24-year-old female in the current study was diagnosed with right surrenal adenoma with a novel pathogenic variant NM_003002.4:c.326A>G on *SDHD* gene (Table 1, Case 18). The same variant was detected in another 82-year-old female who has no relation with the abovementioned 24-year-old female (Table 1, Case 17). Another *SDHD* pathogenic variant was detected in a 30-year-old male who had right surrenal adenoma (Table 1 case 16). *SDHD* and *SDHB* genes were reported as the most important causative genes of hereditary PGL/PCC in Asia when patients are tested with multi-gene NGS panel (21). In a case from Turkey, an 81-year-old female was reported with multinodular goiter and essential hypertension had NM_003002.3:c.325C>T (Gln109Term) pathogenic variant in *SDHD* gene (22).

**Figure 1:**
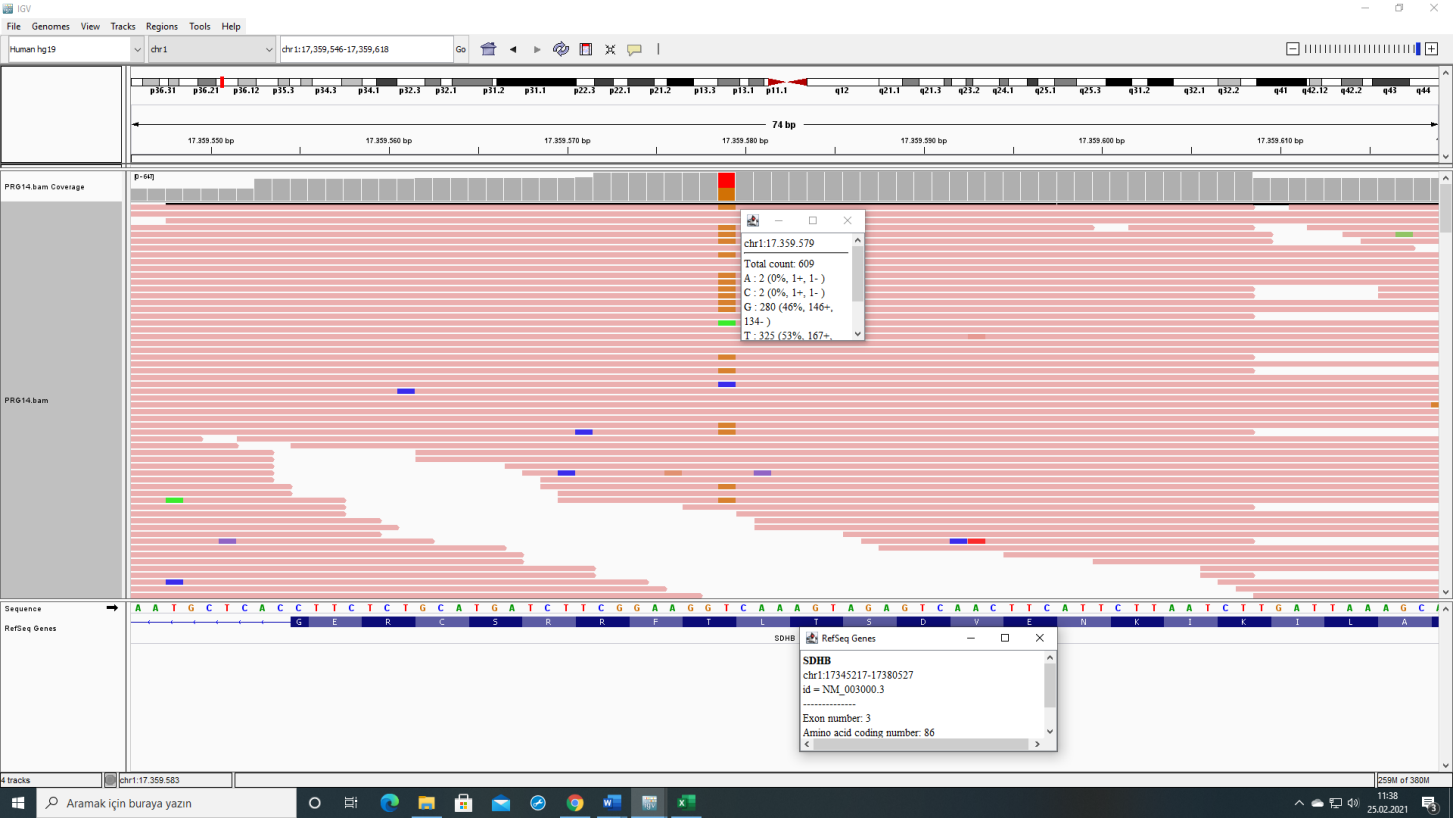
IGV (Integrative Genomics Viewer) image of case 14 showing NM_003000.3:c.262A>C novel variant in *SDHB* gene.

Two different novel pathogenic *SDHB* variants were detected in four cases in our study. All these four cases involved young patients who were not related with each other. Distinct differences were reported in the clinical and histopathological characteristics between genetic variants in *SDHB* (23). Our two nonrelated cases had the same novel pathogenic variant (NM_003000.3:c.262A>C) in *SDHB* gene with different clinical presentations (Table 1, cases 14 and 15) (Figure 1). 58 tumor samples (55 PGL, including 45 head and neck PGL, 2 PCC, 1 GIST) were analysed in a study, and pathogenic variants in 50 patients (22 (13%) *SDHB*, 1 (3.2%) *SDHC* and 27 (57%) *SDHD*) were detected (24). We analyzed germline variants in this study, and the frequencies of the pathogenic variants were 5.3% and 4% for *SDHB* and *SDHD* genes, respectively.

In our study, *SDHB* carriers had no metastasis; only one case had hypertrophic cardiomyopathy, additively (Table 1, Case 15). Metastatic disease occurs in 2–23% of all paraganglioma cases and is a major cause of mortality (25). *SDHB* carriers were reported to have the highest risk for metastasis (26). Genetic counseling was given to these *SDHB* cases and their relatives for future risks, and they have been considered for clinical follow-up.

For *SDHD* carriers, only pathogenic variants inherited from the father will cause disease, due to maternal imprinting with silencing of the maternal allele occurs for *SDHD* (27). Three *SDHD* carriers were identified in our study, paternal analysis could not be planned due to not being alive.

*KIF1B* and *TMEM127* were also included in our targeted gene panel, but any pathogenic/likely pathogenic variant was not detected in these genes. *KIF1B* is a rare reason for pheochromocytoma and *TMEM127* is a susceptibility gene for PGL/PCC. We analyzed the germline variants in the current study. Probably, analyzing somatic variants would support the detection of *KIF1B* and *TMEM127* variants, too.

## Conclusion

To our knowledge, this is the first study from Turkey, analyzing germline *KIF1B, RET, SDHB, SDHD, TMEM127*, and *VHL* variants together. A study from Turkey reported 18 *RET* C634G mutations in 88 individuals (28), while another study reported *VHL* mutations in two pheochromocytoma patients (29). On the other hand, our study has limitations, especially the targeted genes in our study. We could not include other paraganglioma genes like *SDHA, SDHC, MAX*, etc., and we did not include *NF1* carrier cases in this study. Further studies with larger cohorts and larger gene panels are needed for determining the prevalence and genotype-phenotype correlations for PGL/PCC patients.
